# Sevoflurane preconditioning protects experimental ischemic stroke by enhancing anti‐inflammatory microglia/macrophages phenotype polarization through GSK‐3β/Nrf2 pathway

**DOI:** 10.1111/cns.13715

**Published:** 2021-08-09

**Authors:** Min Cai, Sisi Sun, Jin Wang, Beibei Dong, Qianzi Yang, Li Tian, Hailong Dong, Shiquan Wang, Wugang Hou

**Affiliations:** ^1^ Department of Psychiatry Xijing Hospital The Fourth Military Medical University Xi'an China; ^2^ Department of Anesthesiology and Perioperative Medicine Xijing Hospital The Fourth Military Medical University Xi'an China; ^3^ The Medical Department of the Emergence Centre of Xi'an Shaanxi China; ^4^ The Department of Anesthesiology Tianjin Institute of Anesthesiology General Hospital of Tianjin Medical University Tianjin China

**Keywords:** anti‐inflammatory polarity, glycogen synthesis kinase‐3β, ischemic stroke, microglia/macrophages phenotype shift, nuclear factor erythroid 2–related factor, sevoflurane preconditioning

## Abstract

**Aims:**

Sevoflurane preconditioning (SPC) results in cerebral ischemic tolerance; however, the mechanism remains unclear. Promoting microglia/macrophages polarization from pro‐inflammatory state to anti‐inflammatory phenotype has been indicated as a potential treatment target against ischemic stroke. In this study, we aimed to assess the effect of SPC on microglia polarization after stroke and which signaling pathway was involved in this transition.

**Methods:**

Mouse primary microglia with SPC were challenged by oxygen‐glucose deprivation (OGD) or lipopolysaccharide (LPS), and mice with SPC were subjected to middle cerebral artery occlusion (MCAO). Then, the mRNA and protein levels of pro‐inflammatory/anti‐inflammatory factors were analyzed. GSK‐3β phosphorylation and Nrf2 nuclear translocation were measured. The mRNA and protein expression of pro‐inflammatory/anti‐inflammatory factors, neurological scores, infarct volume, cellular apoptosis, the proportion of pro‐inflammatory/anti‐inflammatory microglia/macrophages, and the generation of super‐oxidants were examined after SPC or GSK‐3β inhibitor TDZD treatment with or without Nrf2 deficiency.

**Results:**

Sevoflurane preconditioning promoted anti‐inflammatory and inhibited pro‐inflammatory microglia/macrophages phenotype both in vitro and in vivo. GSK‐3β phosphorylation at Ser9 was increased after SPC. Both SPC and TDZD administration enhanced Nrf2 nuclear translocation, reduced pro‐inflammatory microglia/macrophages markers expression, promoted anti‐inflammatory markers level, and elicited a neuroprotective effect. Nrf2 deficiency abolished the promoted anti‐inflammatory microglia/macrophages polarization and ischemic tolerance induced by TDZD treatment. The reduced percentage of pro‐inflammatory positive cells and super‐oxidants generation induced by SFC or TDZD was also reversed by Nrf2 knockdown.

**Conclusions:**

Our results indicated that SPC exerts brain ischemic tolerance and promotes anti‐inflammatory microglia/macrophages polarization by GSK‐3β‐dependent Nrf2 activation, which provides a novel mechanism for SPC‐induced neuroprotection.

## Graphical abstract

SPC promoted microglia/macrophages polarization toward to anti‐inflammatory phenotype in vitro and in vivo. Both SPC and TDZD administration enhanced Nrf2 nuclear translocation.SPC promoted microglia/macrophages toward to anti‐inflammatory phenotype shifting via GSK‐3β‐dependent Nrf2 activation.

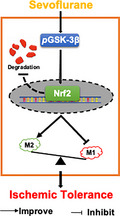

## 1 INTRODUCTION

Cerebral ischemia/reperfusion (I/R) during cardio‐cerebral surgery has serious adverse effects on patient prognosis.^1^ Unfortunately, few therapies for the prevention and treatment of perioperative ischemic stroke have been clinically approved. Therefore, more therapeutic strategies for perioperative stroke are urgently needed. Sevoflurane preconditioning (SPC) results in tolerance against subsequent experimental cerebral I/R damage in vitro and in vivo.^2^, ^3^, ^4^, ^5^ However, the exact molecular and subcellular mechanisms underlying this volatile anesthetic's neuroprotective property are still unclear.

One of the most important pathophysiological features of I/R injury is the inflammatory responses in the central nervous system (CNS).^6^ Although the exact mechanism is not completely understood, the activation of microglia/macrophages seems to be a characteristic process during I/R‐induced excessive brain inflammation.^7^ Microglia/macrophages have particular properties suitable for mediating cellular inflammatory responses during ischemia.^8^, ^9^, ^10^ Among these properties, the switching between the pro‐inflammatory and the anti‐inflammatory microglia phenotype is crucial in regulating inflammatory/anti‐inflammatory gene expression and indenting excessive brain damage.^10^, ^11^, ^12^ Promoting microglia and infiltrated macrophages toward anti‐inflammatory phenotype, which subsequently increased neurogenesis and angiogenesis and remodeled neuronal circuity, could be a target of neuroprotective approaches, including preconditioning stimuli, to produce protective effects against cerebral ischemic damage.

As a multifunctional serine/threonine kinase, glycogen synthase kinase‐3β (GSK‐3β) is highly active in resting cells and usually inhibits multiple downstream pathways. GSK‐3β drives a cascade of signaling pathways, including inflammatory responses in the brain.^13^, ^14^, ^15^ This enzyme participates in the production of pro‐inflammatory factors, while pharmacological or genetic inhibition of this kinase could act as a molecular brake to limit the brain inflammatory response.^15^, ^16^ Accumulating evidence has demonstrated the major role of GSK‐3β inhibition in preventing neuronal death, including its feature in inducing the brain ischemic tolerance of SPC.^14^, ^17^, ^18^ The phosphorylation of GSK‐3β at Ser9, which indicates an inactive status, enables cells to become resistant to various pathophysiological injuries.^19^ However, to our knowledge, the underlying mechanism of GSK‐3β that governs the anti‐inflammatory microglia/macrophages polarization processes caused by SPC after ischemic stroke is still unknown.

In addition to Kelch‐like ECH‐associated protein 1 (Keap1), GSK‐3β has also been identified as another upstream regulator of Nrf2.^20^, ^21^ GSK‐3β could phosphorylate numerous Ser residues in the Neh6 domain of Nrf2, which overlap with an SCF/β‐TrCP destruction motif to promote Keap1‐independent Nrf2 degradation.^22^, ^23^ Nrf2 acts as a “master regulator” in response to oxidative electrophilic stress and chemical insults. It is also a major modulator factor associated with the shift of the anti‐inflammatory/pro‐inflammatory microglia/macrophages phenotype in response to cerebral I/R injury.^12^, ^24^, ^25^ Recent studies have shown that GSK‐3β inhibition‐induced Nrf2 activation plays a crucial role in protecting organs from I/R injury.^18^, ^22^, ^26^ However, whether SPC promoted microglia/macrophages toward anti‐inflammatory phenotype and thereby induced ischemic tolerance through this signaling pathway is still unclear.

In the present study, we used oxygen‐glucose deprivation (OGD)/lipopolysaccharide (LPS) stimulation in primary mouse microglia culture and a transient focal cerebral I/R mouse model to investigate the role of GSK‐3β/Nrf2‐dependent anti‐inflammatory microglia/macrophages phenotype polarization in SPC‐induced neuroprotection.

## 2 MATERIALS AND METHODS

### 2.1 Animals

#### 2.1.1 Animals

All animal‐related procedures were approved by the Ethics Committee for Animal Experimentation of the Fourth Military Medical University (Xi'an, China) and proceeded in accordance with the ARRIVE Guidelines for the Care and Use of Laboratory Animals.^27^ Moreover, the randomization and analgesia procedures were performed in compliance with the ARRIVE guidelines. A randomized number table was used for animal randomization. The analgesia protocol involved postoperative administration of meloxicam (*i*.*p*.) at 0.2 mg/kg body weight and was followed by administration of 0.05 mg/kg body weight for 3 consecutive days postoperation.

Male C57BL6j mice between 8 and 10 weeks old (25–30 g) were purchased from the Animal Laboratory of the Fourth Military Medical University, Xi'an, China. The mice were housed individually and kept on a 12 h alternating light and dark cycle at 20–25°C and 60% humidity with freely available water and food for at least 1 week prior to treatment or surgery. The sample size was based on our previous study; however, the formal statistical power analysis was not used to guide the sample size of this study.^3^ The number of animals used and their suffering were minimized in this study.

#### 2.1.2 Experimental protocols

##### Experiment 1

Analysis of the mRNA expression of pro‐inflammation and anti‐inflammation microglia/macrophages genes (tumor necrosis factor‐α, TNF‐α; interleukin‐1β, IL‐1β; inducible nitric oxide synthase, iNOS, CD‐206, YM1/2, arginase‐1) in vitro.

Two in vitro models were applied in this experiment: OGD and LPS stimulation. The primary cortical microglia received different treatments as follows: control, OGD, SPC + OGD, TDZD + OGD or control, LPS, and sevoflurane SPC + LPS (*n* = 6 per group). After different treatments were confirmed, mRNA levels were measured. Additionally, to further examine the change of Nrf2 and GSK‐3β directly in microglia, protein from control, OGD and SPC + OGD, and TDZD + OGD groups were collected and Nrf2 expressed in nuclear, GSK‐3β phosphorylated at Ser‐9 were analyzed by Western blot (*n* = 4 per group).

##### Experiment 2

Examination of anti‐inflammatory microglia/macrophages polarization and GSK‐3β phosphorylation after SPC and determination of the role of GSK‐3β in the neuroprotective effect and the promotion of microglia/macrophages phenotype induced by SPC.

Mice were randomly allocated to three groups (*n* = 6 per group); (1) Control, (2) SPC + control, (3) I/R and SPC + I/R. The expression of pro‐inflammatory and anti‐inflammatory factors was analyzed seven days after reperfusion by enzyme‐linked immunosorbent assays (ELISAs). Additionally, the expression of iNOS and arginase‐1 in microglia was examined by immunofluorescence staining. The phosphorylation at Ser9 and total protein expression of GSK‐3β were evaluated via Western blots 2 h after reperfusion (*n* = 4 per group). Moreover, the GSK‐3β inhibitor 4‐benzyl‐2‐methyl‐1,2,4‐thiadiazolidine‐3,5‐dione (TDZD, 1 mg/kg in 10% DMSO, *i*.*p*., 1.5 h following reperfusion) was administered based on a previously reported procedure.^28^ Mice were randomly divided into five groups as follows: control, SPC (SPC + I/R), I/R + vehicle, and TDZD + I/R (*n* = 8 per group). The neurological outcomes, infarct volume, and mRNA levels of pro‐inflammatory/anti‐inflammatory microglia phenotype markers were assessed 7 days after reperfusion. An additional cohort of mice was used to measure apoptotic cell death through terminal deoxynucleotidyl transferase deoxyuridine triphosphate‐biotin nick‐end labeling (TUNEL) staining at 72 h after reperfusion (*n* = 5 per group).

##### Experiment 3

Verification of the downstream of GSK‐3β that regulates the anti‐inflammatory microglia/macrophages phenotype polarization induced by SPC.

Mice were randomly divided into the control, control + SPC, control + TDZD, I/R + vehicle, SPC + I/R, and TDZD + I/R groups, and Nrf2 nuclear translocation was assessed using Western blot (*n* = 4 per group). Moreover, an adeno‐associated virus (AAV) shRNA method was used to induce Nrf2 deficiency (AAV‐Nrf2) in this experiment 72 h before MCAO challenge. Mice were divided into the following groups: control, I/R, TDZD + I/R, TDZD + AAV‐Nrf2, and TDZD + AAV‐GFP (control of AAV‐Nrf2) (*n* = 8 per group). The neurological disorder, infarct size, and mRNA levels of pro‐inflammatory/anti‐inflammatory microglia/macrophages marker genes were assessed seven days after reperfusion. TUNEL staining was used to measure apoptotic cell death 72 h after reperfusion in an additional cohort of mice (*n* = 5 per group).

##### Experiment 4

Establishment of dihydroethidium (DHE) oxidation staining and flow cytometry analysis.

Given the crucial role of Nrf2 in regulating antioxidant mechanisms, the DHE oxidation staining method was also used to demonstrate the effect of the GSK‐3β/Nrf2 signaling pathway in SPC. To further analyze the effect of this pathway on the proportion of pro‐inflammatory and anti‐inflammatory microglia/macrophages in SPC‐induced ischemic penumbra, flow cytometry analysis was performed. The mice were divided into the following groups: control, I/R, SPC + I/R, TDZD + I/R, TDZD + AAV‐Nrf2, and TDZD + AAV‐GFP (*n* = 3 per group). The two experiments mentioned above were performed according to previous studies.

#### 2.1.3 Cell culture of mouse primary cortical microglia

Culture of mouse primary cortical microglia was obtained from 24‐h old C57BL/6 newborn pups.^29^ Briefly, the entire brain of mouse was put in ice‐pretreated D‐hanks solution, and then, the meninges and other noncortical tissue were separated. The entire cortex was harvested and digested with 0.25% trypsin (Invitrogen) at 37°C for 7 min, followed by the supplement of DMEM/F12 (Invitrogen) in 10% FBS to stop the digestion. After fully dissociating the cortices with pipettes, the cell suspension was subsequently filtered with a 70‐μm‐diameter mesh. Then, the cells were transferred to a 75 cm^2^ poly‐lysine (PLL, Sigma)–coated flask and incubated at 37°C with 5% CO_2_. About 50% of the culture media was replaced twice per week. After 10 days of culture, primary microglia were collected by shaking the flask for 2 h at 200 rpm and subsequently seeded onto PLL‐precoated new plates for following experiments.

#### 2.1.4 Sevoflurane preconditioning in vitro

The procedure of sevoflurane preconditioning in vitro was based on a previous publication.^30^ Briefly, the primary microglia were placed in an incubator chamber (Billups‐Rothenberg, San Diego, CA), which was flushed for 5 min with 2.5% sevoflurane in the carrier gas of (95% air‐5% CO_2_), and then, the incubator chamber was sealed at 37°C for 1 h. An anesthetic gas analyzer was used to monitor the concentration of sevoflurane in the chamber.

#### 2.1.5 Oxygen and glucose deprivation

The OGD was performed as reported previously.^4^ Primary microglia cells were plated in DMEM with 10% fetal bovine serum, streptomycin (100 μg/ml), and penicillin (100/units) at 37°C in 5% air. During OGD operation, the medium of culture was switched to serum‐ and glucose‐free Dulbecco's modified Eagle's medium and placed in a modular incubator chamber, which was flushed with a mixture of 95% N_2_ and 5% CO_2_ at the rate of 3 L/min at room temperature for 30 min. Control cultures were incubated for the same period of time in a humidified atmosphere of 95% air and 5% CO_2_ at 37°C. After 4‐h challenge, microglial cells were removed from the anaerobic chamber, and the medium of culture was replaced by Dulbecco's modified Eagle's medium containing 10% fetal bovine serum. The generation of reperfusion insult was confirmed by maintaining cells in a humidified 5% CO_2_ incubator for an additional 24 h at 37°C.

#### 2.1.6 LPS stimulation in vitro

The administration of LPS was performed according to the previously reported paradigm.^31^ 24 h before LPS supplement, DMEM/10% FBS was replaced by DMEM/1% FBS. The primary microglial cells were then stimulated with LPS from Escherichia coli, serotype 055:B5 (Sigma, Buchs, Switzerland) with a concentration of 20 μg/ml in DMEM/1% FBS for 2 h. For the control group, cells were only treated with PBS in DMEM/1% FBS instead of LPS.

#### 2.1.7 Cell viability assay

Cell viability was examined by 3‐(4,5‐dimethyl‐2‐thiazolyl)‐2,5‐diphenyl‐2‐H‐tetrazolium bromide (MTT) analyzing kit.^32^ Microglia were cultured 15 × 10^3^ cells per well in 96‐well tissue plates and subjected to various treatments described earlier. At the end of the culture period, cells were washed with PBS, and MTT was added to each well for a 4‐h incubation at 37°C according to the manufacturer's instruction. Then, the medium was switched to dimethyl sulfoxide (Sigma‐Aldrich). The optical density (OD) at 490 nm was measured using a Universal Microplate Reader (Elx 800, Bio‐TEK Instruments Inc., USA). Cell viability results were presented as percentage changes from the baseline value obtained in the control group.

#### 2.1.8 Assessment of lactate dehydrogenase (LDH) release

To examine cell cytotoxicity, LDH released from the injured cells into the culture medium after OGD/LPS stimulation was analyzed by LDH assay kit (Jiancheng Bioengineering Institute, Nanjing, China) following the manufacturer's instructions. LDH leakage was calculated as the percentage of LDH released into the medium out of total LDH activity (LDH in both medium and cells), which is LDH released (%) = (LDH activity in the medium/total LDH activity) × 100%. Cultures without OGD/LPS treatment (control group) served as a baseline for LDH release.

#### 2.1.9 Sevoflurane preconditioning in vivo

Sevoflurane preconditioning of the mice was achieved by inhaling 97% O_2_ containing 2.5 vol% sevoflurane 1 h a day for 5 consecutive days. Mice in the control group inhaled 97% O_2_ without sevoflurane following the same schedule. After the final treatment and a 24‐h washout period, mice were subjected to MCAO surgery.

#### 2.1.10 Arterial blood gas measurement

Five additional mice in each group were used to determine the amount of arterial blood gas. About 0.2 ml blood of each mouse was taken, respectively, from the femoral artery at the end of the subject's last exposure to sevoflurane or oxygen. Samples were analyzed immediately using the OMNI Modular System (Rapidlab 1260, Bayer HealthCare, Uxbridge, United Kingdom).

#### 2.1.11 Transient middle cerebral artery occlusion model

Cerebral I/R injury was induced by a transient middle cerebral artery model in mice as described previously. In brief, after an overnight fast, animals were anesthetized by 3% sevoflurane for induction and 2.5% for surgery. After the right carotid arteries were dissected out, an intraluminal 6‐0 nylon monofilament with a round tip was inserted from the right common carotid artery to the right middle cerebral artery. Following 1 h of transient occlusion, the filament was withdrawn to allow reperfusion. The temporal temperature was maintained at 37 ± 0.5°C by a thermostatic blanket and a lamp. Sham‐operated mice in the control group were subjected to the same surgical procedure but without inserting the filament.

A laser Doppler flowmeter (PeriFlux 5000; Perimed AB, Sweden) was placed on the skull's dorsal surface (caudal 2 mm and lateral 5 mm to bregma) before, during, and after the operation to quantitate the regional cerebral blood flow (rCBF). Mice were excluded from the final analysis if their rCBF did not fall below 20% of baseline during occlusion or recover over 80% during reperfusion. Moreover, if the MCAO procedure could not be completed within 10 min, the corresponding data were also discarded.

#### 2.1.12 Neurobehavioral evaluation and infarct size assessment

Neurobehavioral outcomes in the mice were assessed in accordance with the Garcia Score Scale by an observer blinded to the animal groups.^33^ Data were expressed as median (interquartile range).

The infarct volume was assessed by TTC staining following standard procedures. Briefly, the mouse was decapitated after the last neurobehavioral test. The brain was rapidly removed and cooled in ice‐cold saline for 5 min. Coronal sections (1 mm) were prepared, immersed in 2,3,5‐triphenyltetrazolium chloride (1%; TTC, Sigma‐Aldrich, St. Louis, MO) for 10 min, and then fixed with 4% paraformaldehyde in 0.01 M PBS (pH 7.4) for 24 h. Images of brain slices were acquired using a digital camera (Canon IXUS 220HS). The infarct volume was calculated by Swanson's method to correct for edema: 100% × (contralateral hemisphere volume − nonlesioned ipsilateral hemisphere volume)/contralateral hemisphere volume.^34^

#### 2.1.13 TUNEL staining for apoptosis assessment

Cellular apoptosis was evaluated 72 h after reperfusion by TUNEL staining. Using the in situ cell death detection kit (Roche, German), TUNEL staining was performed on paraffin‐embedded sections based on an established protocol. The positive cells were acquired from areas in the ischemic penumbra using a 40× objective lens, and the number of TUNEL positive cells was expressed as number per 100 br^2^. The same paradigm employed in past research was used to define the ischemic penumbra area in this study.^35^

#### 2.1.14 RNA isolation and quantitative PCR

Total RNA was extracted from primary microglia or brain penumbra using Trizol reagent (Invitrogen, USA) following the manufacturer's instructions. Isolated RNA was reverse transcribed into cDNA with a cDNA synthesis kit (Invitrogen, USA) in strict accordance with standard procedures. Quantitative PCR was performed using synthetic primes and SYBR Green (Invitrogen, USA) with an IQ5 Detection System (Bio‐Rad). The PCR cycles began with template denaturing at 95°C for 5 min, followed by 40 cycles of 95°C for 10 s, 60°C for 20 s, 72°C for 20 s, and 78°C for 20 s.

All primers used in this study were listed in Table 1.

**TABLE 1:** The primers of anti‐inflammation and pro‐inflammation factors used in this study

Gene Name	Sense (5′‐3′)	Anti‐sense (5′‐3′)
GAPDH	AACTTTGGCATTGTG GAAGG	GGATGCAGGGATGATGTTCT
TNF‐α	GCTGAGCTCAAACCCTGGTA	CGGACTCCGCAAAGTCTAAG
IL−1β	TGTGAAATGCCATTTGA	GGTCAAAGGTTTGGAAGCAG
iNOS	CCCAGAGTTCCAGCTTCTGG	CCAAGCCCCTCACCATTATCT
CD−206	CTTCGGGCCTTTGGAATAAT	TAGAAGAGCCCTTGGGTTGA
YM1/2	CAGGGTAATGAGTGGGTTGG	CACGGCACCTCCTAAATTGT
Arg1	CTGGTCGGTTTGATGCTA	TGCTTAGCTCTGTCTGCTTTGC

#### 2.1.15 Western blot

At 30 min and 2 h after reperfusion, mice from each group (*n* = 5) were euthanized. Brain tissues corresponding to the ischemic penumbra were harvested as previously described. The tissues were homogenized in ice‐cold RIPA lysis buffer containing 1% phenylmethanesulfonylfluoride (Beyotime, Nantong, China). To examine Nrf2 nuclear translocation, nuclear protein was exacted with a Nuclear Extraction Kit (Pierce Biotechnology, USA). The concentration of protein was measured by the Bradford method with an available kit (Beyotime, Nantong, China). The primary antibodies were listed as following: anti‐GSK‐3β and *p*‐GSK‐3β (Ser‐9) (1:1000, respectively; Cell signaling technology), anti‐Nrf2 (1:500; Abcam), anti‐glyceral‐dehyde‐3‐phosphate dehydrogenase (1:2000; Abcam), and histone‐3 (1:500; Signal way antibody). After the membranes were incubated with appropriated secondary antibodies, the blots were immersed in an enhanced chemiluminescent reagent and then exposed to enhanced chemiluminescent‐Hyperfilm (Amersham Biosciences) to visualize specific protein bands.

#### 2.1.16 Immunofluorescence staining

For immunofluorescence staining, brain sections were washed with phosphate‐buffered saline containing 1% Triton for 3 times and then incubated with goat anti‐IBA1 antibody (1:300, Abcam), rabbit anti‐iNOS antibody (1:100, Genetex), or rabbit anti‐Arg1 antibody (1:200, Genetex) for 24 h at 4°C. After being washed with PBS for 3 times, the sections were incubated with CY3‐labeled goat anti‐rabbit and FITC‐labeled goat anti‐rat secondary antibodies (1:1000 for both, Millipore) at room temperature for 2 h. Lastly, sections was incubated with the nuclei marker DAPI (1 ng/u, Sigma) for 5 min at room temperature. Fluorescent signals were detected using a confocal fluorescence microscope (Flv100i, Olympus).

#### 2.1.17 Construction and transfection of Nrf2‐shRNA

The Nrf2‐shRNA (AAV9‐Nfe2l2‐RNAi, 2.97 × 10^12^ v.g./ml) and control AAV9‐GFP were purchased from GeneChem Co., Ltd. (Shanghai, China; No. GIDV0166296). The target sequence was 5′‐CGCTGAGTACTTCGAAATGTC‐3′. AAV‐Nrf2 or AAV‐GFP transfection was confirmed by intracerebroventricular injection. The stereotaxic coordinate location of the lateral cerebral ventricle was 0.4 mm posterior to the bregma, 1.0 mm lateral to the midsagittal line, and 2.0 mm deep from the cranial surface. At 72 h after injection, efficacy of the shRNA was determined by Western blot. As shown in Figure S3, AAV‐Nrf2‐shRNA but not AAV‐GFP reduced the expression of Nrf2 protein.

#### 2.1.18 Dihydroethidium oxidation staining

After deep anaesthetization, the mice were decapitated, and the brains were quickly removed and placed onto a base mold surrounded with TBS Tissue Freezing medium. After being placed in dry ice for 15 min, the brains were cut on a cryostat into 20‐μm‐thick sections. Sections were then incubated with DHE working solution (10 mM, Beyotime) at 37°C for 30 min and covered with 24‐mm square glass coverslips.

The images of DHE oxidation were captured by confocal fluorescence microscopy (Flv100i, Olympus). The quantification of DHE‐positive cells was performed in accordance with the method reported by Dugan et al.^36^

#### 2.1.19 Statistical analysis

GraphPad Prism for Windows (version 7.0) was used to conduct the statistical analyses. The neurological score was expressed as the median with the interquartile range and analyzed by a nonparametric Kruskal‐Wallis test followed by a Bonferroni‐corrected Mann–Whitney *U* test. The other data are presented as the mean ± S.D. Bartlett's test for homogeneity of variance was performed to ensure a normal distribution of data, while *p* > 0.05 indicated an acceptable homogeneity of variance for subsequent one‐way ANOVA analyses. The differences between the two groups were detected with Tukey's post hoc test. Two‐tailed values of *p* < 0.05 were considered statistically significant.

## 3 RESULTS

### 3.1 SPC promoted mouse primary microglia polarization into the anti‐inflammatory microglia/macrophages phenotype, increased Nrf2 nuclear expression and GSK‐3β phosphorylation against OGD

**FIGURE 1:**
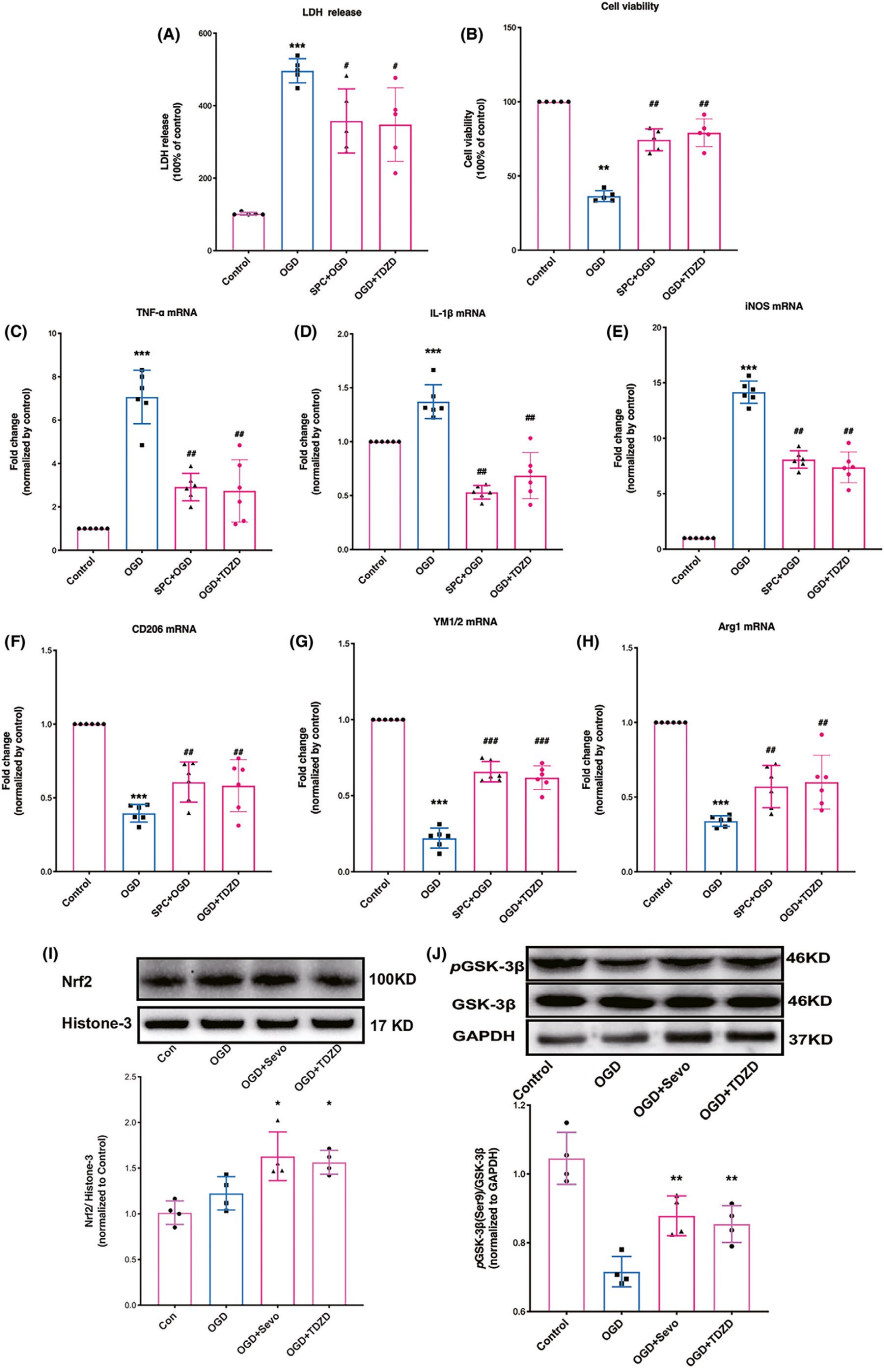
SPC and TDZD shifted microglia/macrophages polarization toward anti‐inflammatory phenotype, increased Nrf2 nuclear expression and GSK‐3β phosphorylation after OGD challenge. (A, B) Effect of SPC and TDZD on cell viability (A) and plasma lactate dehydrogenase (LDH) release levels in primary cortical microglia after OGD (B). (C–E) The mRNA expression of pro‐inflammatory cytokines (TNF‐α, IL‐1β, and iNOS) 24 h following OGD. (F–H) The mRNA levels of anti‐inflammatory cytokines (CD‐206, YM1/2, arginase‐1) 24 h following OGD. (I) Western blot analysis for Nrf2 nuclear expression. (J) Western blot analysis for the phosphorylation of GSK‐3β. (*n* = 4). *p < 0.05, **p < 0.01, ***p < 0.001 versus the control group; #*p* < 0.05, ##*p* < 0.01, ###*p* < 0.001 versus OGD. One‐way ANOVA with Tukey's post hoc test was used for statistical analysis. IL‐1β, Interleukin‐1β; iNOS, inducible nitric oxide synthase; OGD, oxygen‐glucose deprivation; Sevo, sevoflurane preconditioning; TNF‐α, Tumor necrosis factor‐α

As illustrated in Figure 1A, OGD increased the lactate dehydrogenase (LDH) release to 496.4 (14.91%) compared to that of the control group (*p* < 0.0001), SPC or TDZD treatment showed a significant effect on the prevention of increased LDH levels induced by OGD [*p* = 0.0046, 0.0026, respectively]. Compared with that in the control group, cell viability in the OGD group was significantly reduced (Figure 1B). In addition, SPC or TDZD treatment was able to significantly restore cell viability [*p* = 0.0037, 0.0017, respectively].

As shown in Figure 1C–E, the pro‐inflammatory factor (TNF‐α, IL‐1β, iNOS) mRNA levels in the OGD group were higher than those in the control group at 24 h after OGD. When mouse primary microglial cells were pretreated with sevoflurane or TDZD, mRNA expression was reduced compared with that of the OGD group (*p* = 0.0023, <0.001, 0.0073, respectively). The mRNA changes of anti‐inflammatory mediators are presented in Figure 1F–H. The expressions of CD206, YM1/2, and arginase‐1 mRNA were reduced in the OGD group as compared with those in the control group (*p* < 0.001, respectively), while SPC or TDZD treatment prevented this reduction (*p* = 0.0019, <0.001, and 0.007, respectively).

As shown in Figure 1I, Nrf2 protein content in the nucleus was increased in the SPC and TDZD‐treated groups, but not in the SPC‐treated control group, as compared to the I/R group (*p* = 0.037 and 0.018, respectively). The level of phosphorylated GSK‐3β in the OGD groups was decreased compared with that of the control group, but this reduction was reversed in the SPC and TDZD‐treated group (Figure 1J, *p *= 0.0074 and 0.0053, respectively).

### 3.2 SPC reduced the pro‐inflammatory factors, increased the anti‐inflammatory cytokines, and protected microglia culture against LPS‐induced injury

**FIGURE 2:**
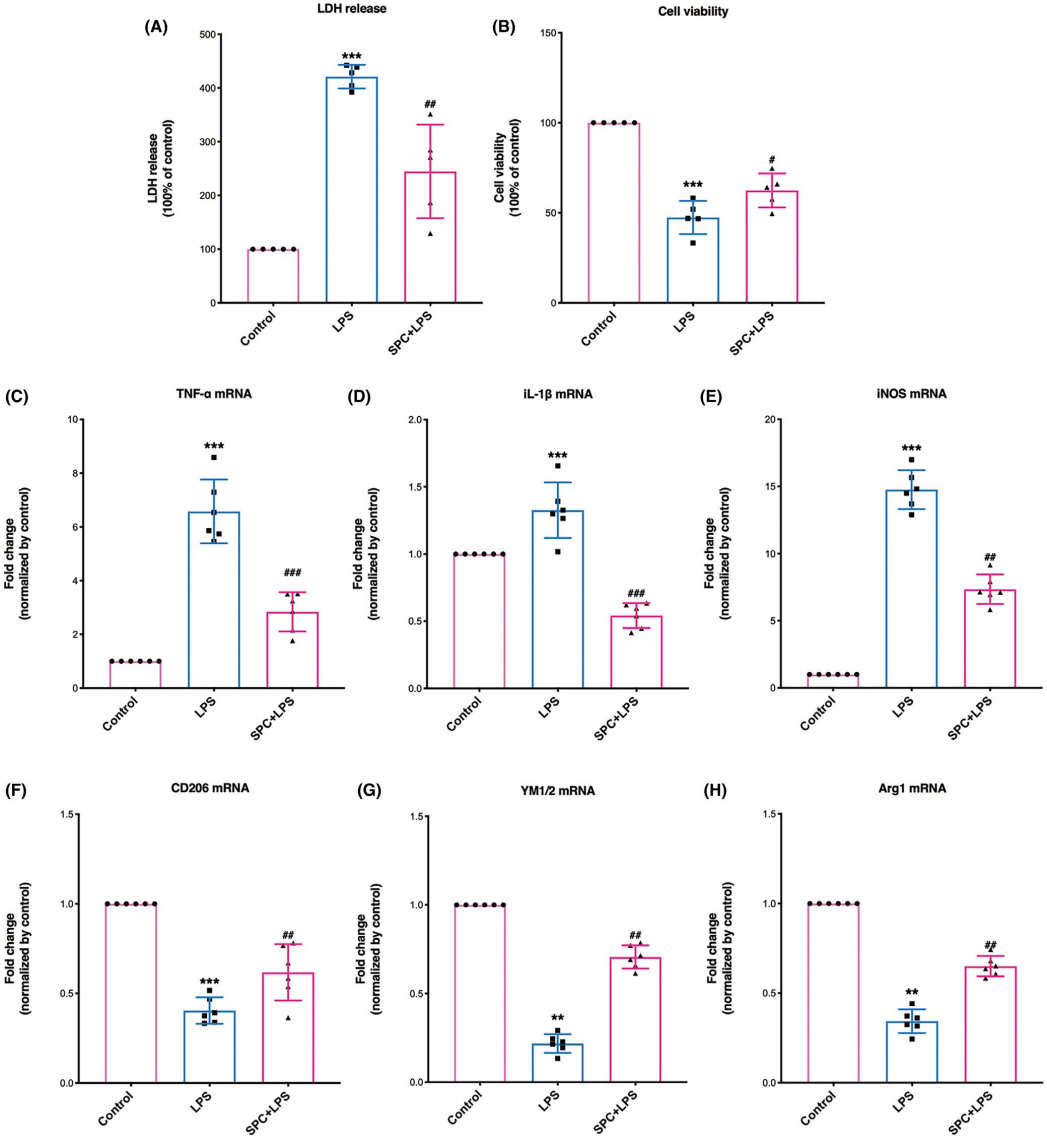
SPC reduced the mRNA expression of pro‐inflammatory cytokines and increased the mRNA levels of anti‐inflammatory cytokines 24 h after LPS stimulation. (A, B) Effect of SPC on cell viability (A) and plasma lactate dehydrogenase (LDH) release levels in primary cortical microglia after LPS (B). (C–E) The mRNA expression of pro‐inflammatory cytokines (TNF‐α, IL‐1β, and iNOS) following LPS. (F–H) The mRNA levels of anti‐inflammatory cytokines (CD‐206, YM1/2, arginase‐1) following LPS. IL‐1β, Interleukin‐1β; iNOS, inducible nitric oxide synthase; LPS, lipopolysaccharide; Sevo, sevoflurane preconditioning; TNF‐α, Tumor necrosis factor‐α. *p < 0.05, **p < 0.01, ***p < 0.001 versus the control group; #*p* < 0.05, ##*p* < 0.01, ###*p* < 0.001 versus OGD/LPS. One‐way ANOVA with Tukey's post hoc test was used for statistical analysis

Moreover, LPS stimulation was also performed in this study to further determine the effect of SPC on polarizing microglia toward the anti‐inflammatory phenotype. The microglial culture was severely damaged by LPS stimulation, which was evident from the increased LDH release (Figure 2A) and decreased MTT (Figure 2B) levels in the LPS group (*p* < 0.001). This impairment was reduced when the microglia cells were pretreated with sevoflurane (*p* = 0.0023 for LDH, *p* = 0.022 for MTT).

Moreover, LPS treatment significantly up‐regulated mRNA levels of pro‐inflammatory marker genes (Figure 2C–E, TNF‐α, IL‐1β, and iNOS), while SPC exerted a significant effect on the inhibition of LPS‐induced elevation of these pro‐inflammatory genes at 24 h after LPS (*p* = 0.0033, <0.001, and 0.0015, respectively). In addition, SPC treatment improved the mRNA levels of anti‐inflammatory cytokine genes (Figure 2F–H, CD206: *p* = 0.0057, YM1/2: *p* = 0.0056, arginase‐1: *p* = 0.0037).

### 3.3 **SPC shifts microglia/macrophages polarization toward anti‐inflammatory phenotype in the Ischemic Hemisphere 7 days after reperfusion**.

Changes in physiologic parameters at the end of the preconditioning operation and various intervals of I/R are summarized in Table S1. No significant differences in pH values, temporal temperatures, or partial pressures of carbon dioxide (P_CO2_) were detected among the groups. As shown in Figure S1, SPC did not alter the regional cerebral blood flow.

**FIGURE 3:**
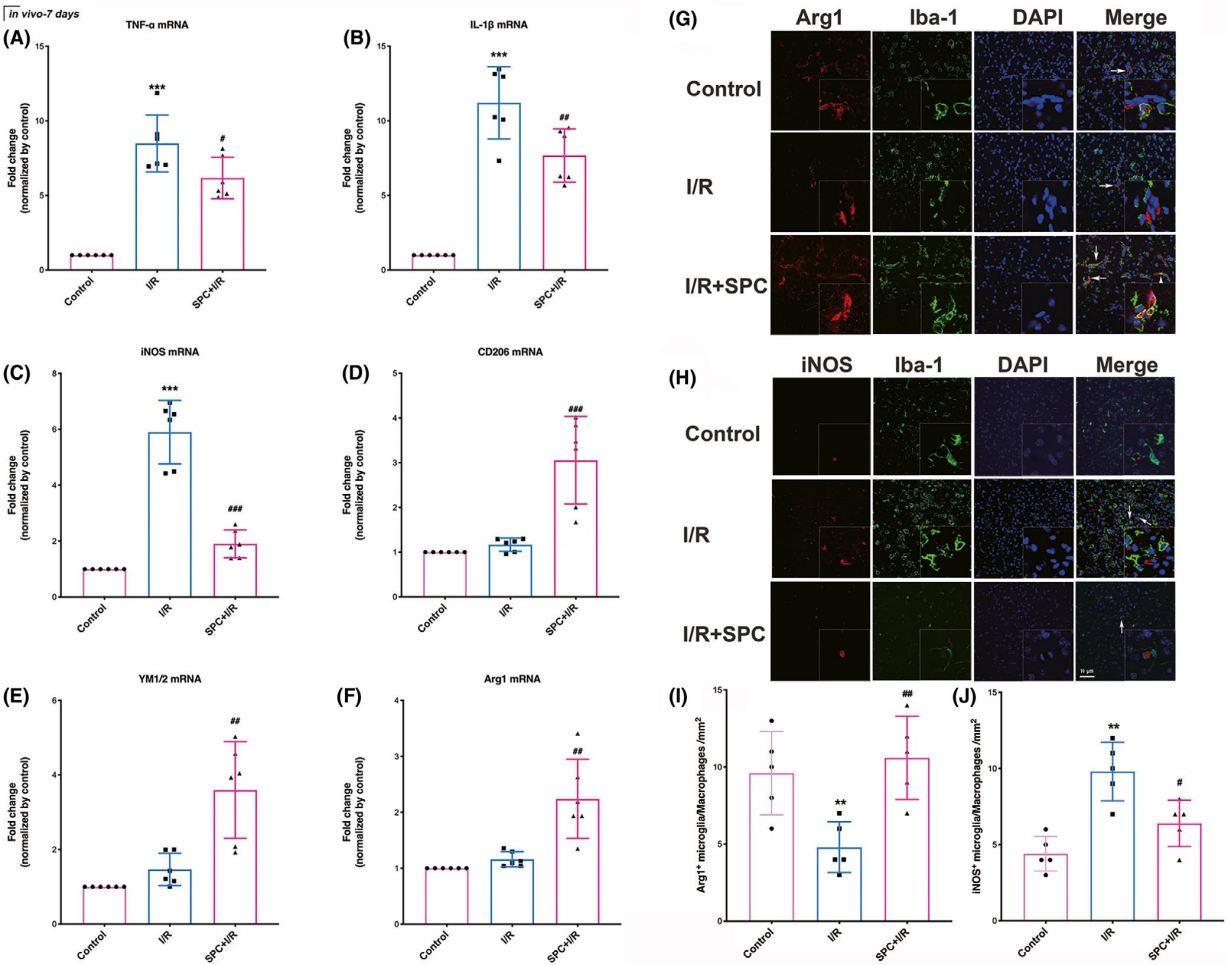
TDZD reduced the mRNA expression of pro‐inflammatory cytokines and increased the mRNA level of anti‐inflammatory cytokines after ischemia/reperfusion. (A–C) The mRNA expression of pro‐inflammatory factors (TNF‐α, IL‐1β, and iNOS) following 7 days of reperfusion. (D–F) The mRNA levels of anti‐inflammatory cytokines (CD‐206, YM1/2, arginase‐1). (G–J) The immunofluorescence staining of iNOS (G) and Arg1 (H) in microglia/macrophages and the quantitative statistical results of iNOS and Arg1‐positive cells in each group (I, J). Scale bar = 10 μm. *p < 0.05, **p < 0.01, ***p < 0.001 versus the control group; #*p* < 0.05, ##*p* < 0.01, ###*p* < 0.001 versus the I/R group. One‐way ANOVA with Tukey's post hoc test was used for statistical analysis. I/R, ischemia/reperfusion; IL‐1β, Interleukin‐1β; iNOS, inducible nitric oxide synthase; Sevo, sevoflurane preconditioning; TNF‐α, Tumor necrosis factor‐α

As presented in Figure 3A–C, the TNF‐α, IL‐1β, and iNOS mRNA levels were increased on day 7 after I/R, while this elevation was reversed by sevoflurane pretreatment (*p* = 0.0202, 0.006, and <0.001, respectively). As shown in Figure 3D–F, I/R did not alter the mRNA expression of anti‐inflammatory mediators (CD206: *p* = 0.6507, YM1/2: *p* > 0.9999, arginase‐1: *p* = 0.7854). However, the mRNA levels of anti‐inflammatory phenotype markers in the SPC‐treated group increased significantly compared with those of the I/R group (*p* = 0.0006, *p* = 0.0013, *p* = 0.0012).

As shown in Figure 3G,I, the number of Arg1 positive microglia in ischemic penumbra increased in the SPC group but not in the I/R group (*p* = 0.0079). In line with this, the number of iNOS positive microglia increased in the I/R group, while SPC treatment reversed this increase (*p* = 0.032, Figure 3H,J).

### 3.4 SPC increased the phosphorylation of GSK‐3β, and supplementation with a GSK‐3β inhibitor reduced cerebral I/R injury

**FIGURE 4:**
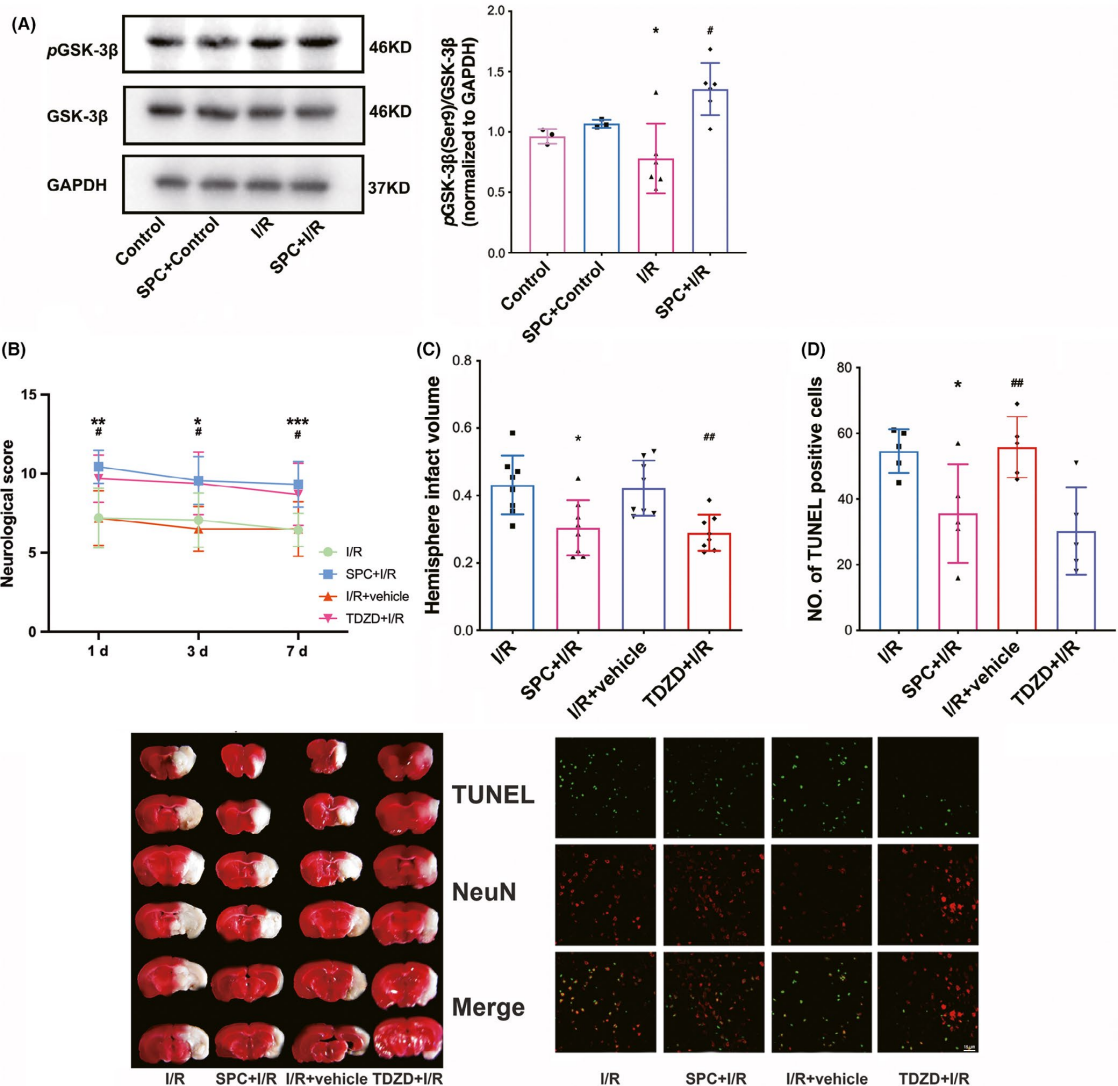
SPC and TDZD treatment increased Nrf2 nuclear translocation; knockdown of Nrf2 reversed the neuroprotection induced by TDZD. (A) Western blot analysis of Nrf2 translocation. (*n* = 4 per group). (B) Nrf2 deficiency prevented the ameliorated neurological manifestations induced at 1, 3 and 7 days after reperfusion. Each symbol presents the score of a single rat. (C) Comparisons of the percentages of infarction size among the control, I/R, TDZD + I/R, TDZD + AAV‐Nrf2 and TDZD + AAV‐GFP groups (*n* = 8). (D) Cellular apoptosis was measured by TUNEL staining in the ischemic penumbra. The panel below is the quantitative statistical results of TUNEL‐positive cells in each group (*n* = 5). Scale bar = 10 μm. *p < 0.05, **p < 0.01 versus the I/R group; #*p* < 0.05 versus the TDZD + I/R group. One‐way ANOVA with Tukey's post hoc test was used for statistical analysis. AAV‐Nrf2, adeno‐associated virus induced Nrf2‐shRNA; I/R, ischemia/reperfusion; TDZD, 4‐benzyl‐2‐methyl‐1,2,4‐thiadiazolidine‐3,5‐dione; TUNEL, terminal deoxynucleotidyl transferase‐mediated 2′‐deoxyuridine 5′‐triphosphate nick‐end labeling

As shown in Figure 4A, GSK‐3β phosphorylation was determined by Western blot analysis. The abundance of phosphorylated GSK‐3β in the I/R groups was reduced compared with that of the control group (*p* = 0.0218), but this reduction was ameliorated in the SPC group (SPC vs. I/R, *p* = 0.0206). SPC did not alter GSK‐3β phosphorylation levels in control mice. Neither I/R nor SPC treatment affected the total GSK‐3β protein expression.

To further verify the role of GSK‐3β in I/R tolerance, we used a GSK‐3β inhibitor, TDZD, in this study. As demonstrated in Figure S2, GSK‐3β phosphorylation was measured by Western blots. The phosphorylation of GSK‐3β at Ser9 was decreased in the vehicle‐treated I/R groups compared with the control group (*p* = 0.0210), but this reduction was ameliorated by TDZD supplementation (TDZD + I/R vs. I/R + vehicle, *p* = 0.0045). Moreover, as shown in Figure S3, the TDZD + SPC group did not further enhance the phosphorylation of GSK‐3β at Ser9.

As shown in Figure 4B, SPC significantly improved the neurobehavioral outcome, which is evident from the neurological scores on day 1 [10.75, (9.00, 12.00)], day 3 [10.00 (7.00, 11.00)], and day 7 [9.50, (7.00, 11.00)] in the SPC + I/R group compared with the I/R group at parallel time points (*p* = 0.0031, 0.0121, and 0.0008, respectively, for each comparison). Consistent with the improvement in neurological outcome, the infarct volume in the SPC + I/R group was smaller than that in the I/R group [Figure 4C, 30.5 (2.9%) vs. 43.1 (3.1%), *p* = 0.0142). Following the administration of TDZD, mice that underwent the MCAO surgery showed better neurobehavioral performance than I/R mice 1, 3 and 7 day(s) after reperfusion (*p* = 0.0171, 0.0241, 0.0219, respectively). Additionally, supplementation with TDZD reduced the brain infarct volume compared with that of the I/R mice [28.9 (1.9%) vs. 43.1 (3.1%), *p* = 0.0053]. No significant difference was detected between the I/R and I/R + vehicle groups.

Neuronal cell apoptosis in the ischemic penumbra is presented in Figure 4D. The number of TUNEL‐positive cells in the SPC group was significantly lower compared with that of the I/R group (*p* = 0.0186). Moreover, the number of TUNEL‐positive cells in the TDZD‐treated group decreased compared to that in the I/R + vehicle group (*p* = 0.0314).

### 3.5 GSK‐3β Inhibition promoted microglia/macrophages shift to the anti‐inflammatory phenotype in the ischemic hemisphere 7 days after reperfusion

**FIGURE 5:**
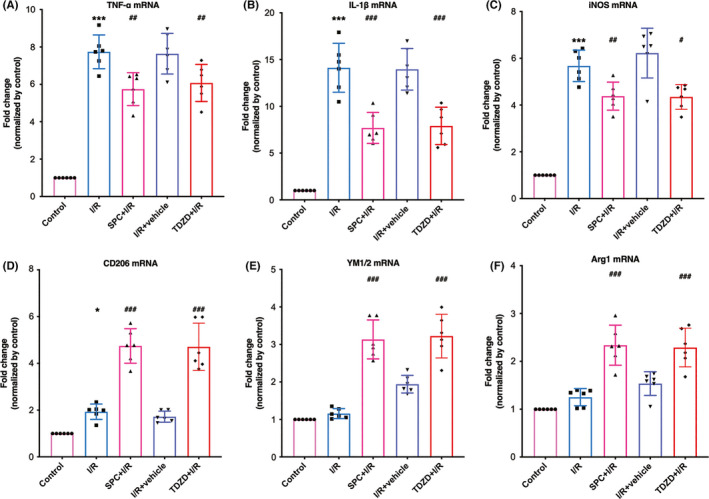
TDZD reduced the mRNA expression of pro‐inflammatory cytokines and increased the mRNA level of anti‐inflammatory cytokines after ischemia/reperfusion. (A–C) The mRNA expression of pro‐inflammatory factors (TNF‐α, IL‐1β and iNOS) following 7 days of reperfusion. (D–F) The mRNA levels of anti‐inflammatory cytokines (CD‐206, YM1/2, arginase‐1). *n* = 6, *p < 0.05, **p < 0.01, ***p < 0.001 versus the control group; #*p* < 0.05, ##*p* < 0.01, ###*p* < 0.001 versus the I/R group. One‐way ANOVA with Tukey's post hoc test was used for statistical analysis. I/R, ischemia/reperfusion; IL‐1β, Interleukin‐1β; iNOS, inducible nitric oxide synthase; Sevo, sevoflurane preconditioning; TNF‐α, Tumor necrosis factor‐α

As shown in Figure 5A–C, the mRNA expression of the pro‐inflammatory cytokines was significantly elevated in the ischemic penumbra but was reduced when mice received SPC. The TDZD treatment also reduced the elevated mRNA expression of these pro‐inflammatory cytokines (TDZD + I/R vs. I/R: *p *= 0.0206, <0.001, 0.0157, respectively). As shown in Figure 5D–F, the mRNA levels of the anti‐inflammatory cytokines in the SPC group were higher than those in the I/R group. TDZD also increased the level of these anti‐inflammatory factors (CD206, YM1/2, arginase‐1: *p* < 0.001).

### 3.6 SPC and TDZD treatment increased nuclear translocation of Nrf2, and Nrf2 deficiency reversed the neuroprotective effect produced by TDZD supplementation

**FIGURE 6:**
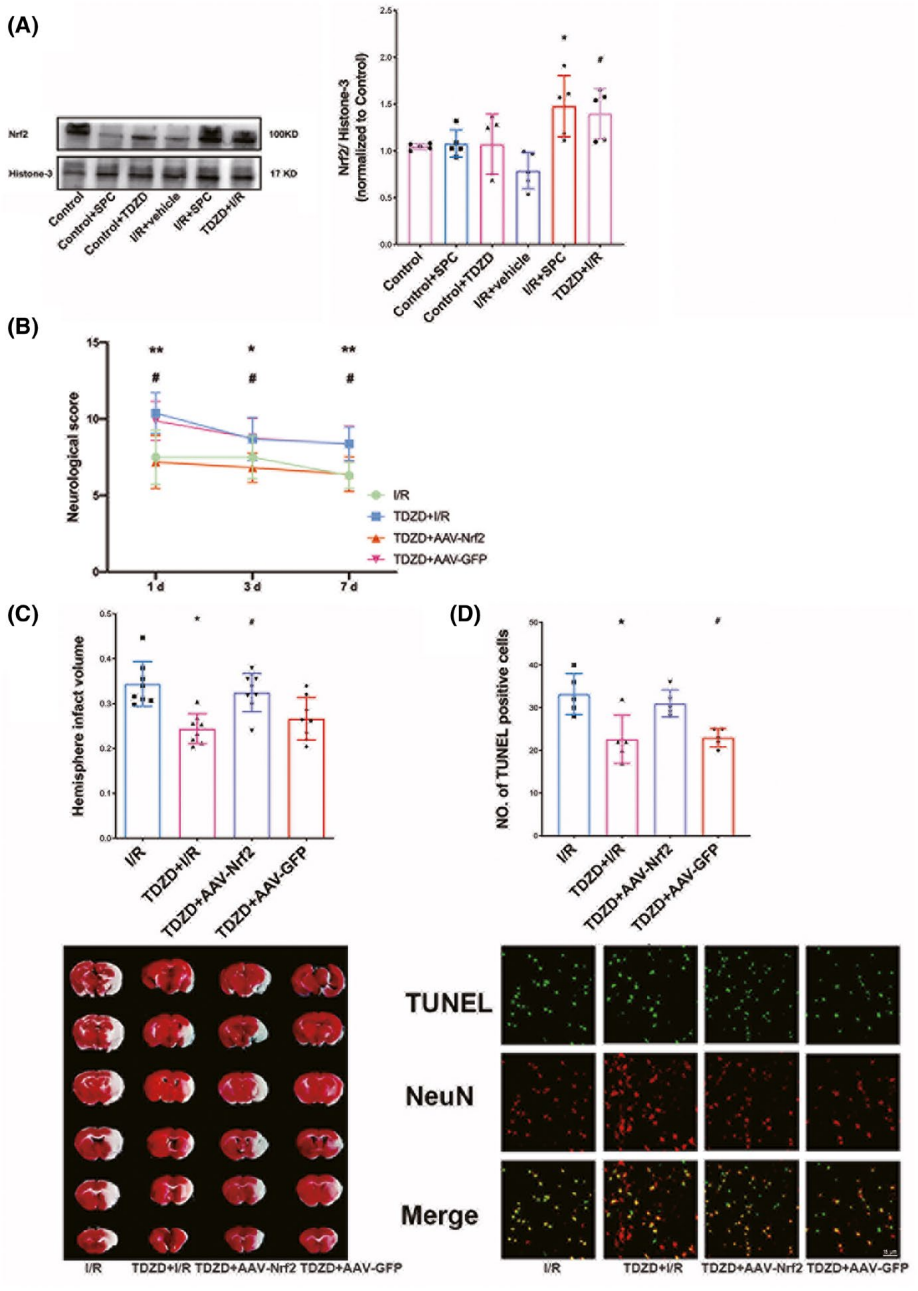
SPC and TDZD treatment increased Nrf2 nuclear translocation; knockdown of Nrf2 reversed the neuroprotection induced by TDZD. (A) Western blot analysis of Nrf2 translocation. (*n* = 6 per group). (B) Nrf2 deficiency prevented the ameliorated neurological manifestations induced at 1, 3 and 7 days after reperfusion. Each symbol presents the score of a single rat. (C) Comparisons of the percentages of infarction size among the control, I/R, TDZD + I/R, TDZD + AAV‐Nrf2, and TDZD + AAV‐GFP groups (*n* = 8). (D) Cellular apoptosis was measured by TUNEL staining in the ischemic penumbra. The panel below is the quantitative statistical results of TUNEL‐positive cells in each group (*n* = 5). Scale bar = 15 μm. *p < 0.05, **p < 0.01 versus the I/R group; #*p* < 0.05 versus the TDZD + I/R group. One‐way ANOVA with Tukey's post hoc test was used for statistical analysis. AAV‐Nrf2, adeno‐associated virus induced Nrf2‐shRNA; I/R, ischemia/reperfusion; TDZD, 4‐benzyl‐2‐methyl‐1,2,4‐thiadiazolidine‐3,5‐dione; TUNEL, terminal deoxynucleotidyl transferase‐mediated 2′‐deoxyuridine 5′‐triphosphate nick‐end labeling

As shown in Figure 6A, Nrf2 protein content in the nucleus was increased in the SPC and TDZD‐treated groups, but not in the SPC‐treated control group, as compared to the I/R group (*p* = 0.0222 and 0.0323, respectively). Nevertheless, SPC failed to change the Nrf2 expression in control mice.

To further determine the relationship between GSK‐3β and Nrf2 under SPC, we applied an Nrf2‐shRNA (AAV‐Nrf2‐shRNA and its control AAV‐GFP) in this experiment to silence the expression of Nrf2. As shown in Figure 6B, TDZD significantly enhanced the neurobehavioral outcome. At the same time, Nrf2 knockdown reversed this improvement, as judged by the neurological scores at 1, 3 and 7 day(s) in the TDZD + AAV‐Nrf2 group compared with the TDZD group at parallel time points (*p* = 0.0193, 0.0219, and 0.0132, respectively, for each comparison). Consistent with the change in neurological outcome, the TDZD + AAV‐Nrf2 group's infract size was larger than that in the TDZD group at 7 days after reperfusion [Figure 6C, 32.51 (1.51%) vs. 24.34 (1.2%), *p* = 0.0049]. However, no significant difference was detected between the I/R and I/R + vehicle groups.

Furthermore, the reduced number of TUNEL‐positive cells in the TDZD group was significantly reversed by introducing Nrf2 knockdown (Figure 6D, *p *= 0.0265).

### 3.7 Knockdown of Nrf2 abolished the promoted anti‐inflammatory microglia/macrophages shift produced by GSK‐3β Inhibition

**FIGURE 7:**
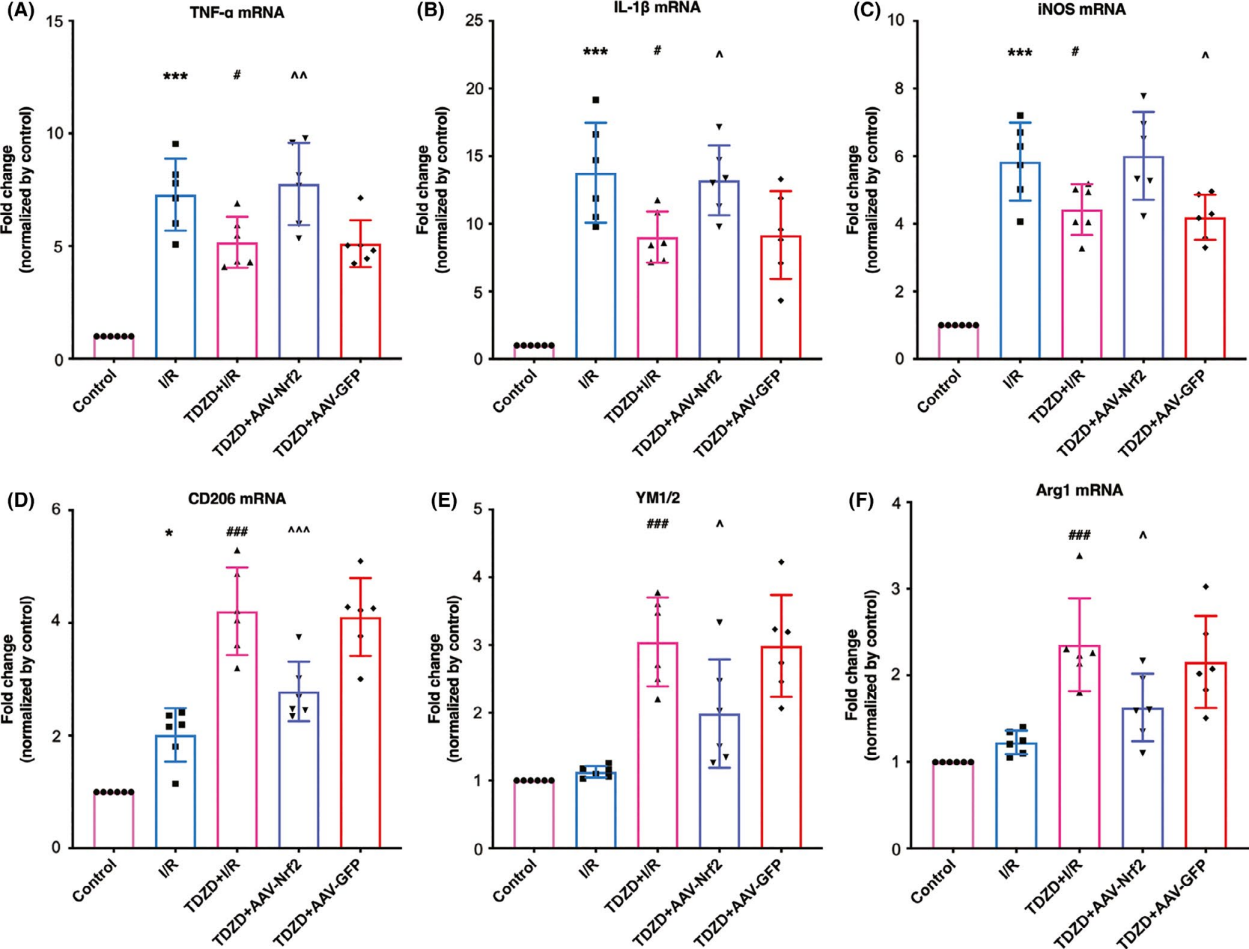
The deletion of Nrf2 reversed the reduced mRNA expression of pro‐inflammatory cytokines and increased the mRNA level of anti‐inflammatory cytokines induced by TDZD administration after ischemia/reperfusion. (A–C) The mRNA expression of pro‐inflammatory cytokines (TNF‐α, IL‐1β, and iNOS) following 7 days of reperfusion. (D–F) The mRNA levels of anti‐inflammatory factors (CD‐206, YM1/2, arginase‐1) 7 days after reperfusion. *n* = 6, *p < 0.05, **p < 0.01, ***p < 0.001 versus the I/R group; #*p* < 0.05, ##*p* < 0.01, ###*p* < 0.001 versus the I/R group; ^*p* < 0.05, ^^*p* < 0.01, ^^^*p* < 0.001. One‐way ANOVA with Tukey's post hoc test was used for statistical analysis. AAV‐Nrf2, adeno‐associated virus induced Nrf2‐shRNA; I/R, ischemia/reperfusion; IL‐1β, Interleukin‐1β; iNOS, inducible nitric oxide synthase; TDZD, 4‐Benzyl‐2‐methyl‐1,2,4‐thiadiazolidine‐3,5‐dione; TNF‐α, Tumor necrosis factor‐α

AAV‐Nrf2 microinjection led to a significant reduction of Nrf2 expression (see Figure S3). As presented in Figure 7A–C, mRNA expression of pro‐inflammatory factors was significantly reduced in the ischemic penumbra of mice treated with TDZD; however, this attenuated expression was terminated by the administration of AAV‐Nrf2 (*p* = 0.0073, 0.0407, 0.0205, respectively). As demonstrated in Figure 7D and E, the mRNA levels of anti‐inflammatory factors were higher in the TDZD group than that in the I/R group, while Nrf2 deficiency prevented this improvement (CD206, YM1/2, and arginase‐1: *p* < 0.001, =0.0150, and 0.0127, respectively).

### 3.8 Knockdown of Nrf2 reversed anti‐inflammatory microglia/macrophages polarization and abolished the inhibition of reactive oxygen species generation produced by TDZD treatment

**FIGURE 8:**
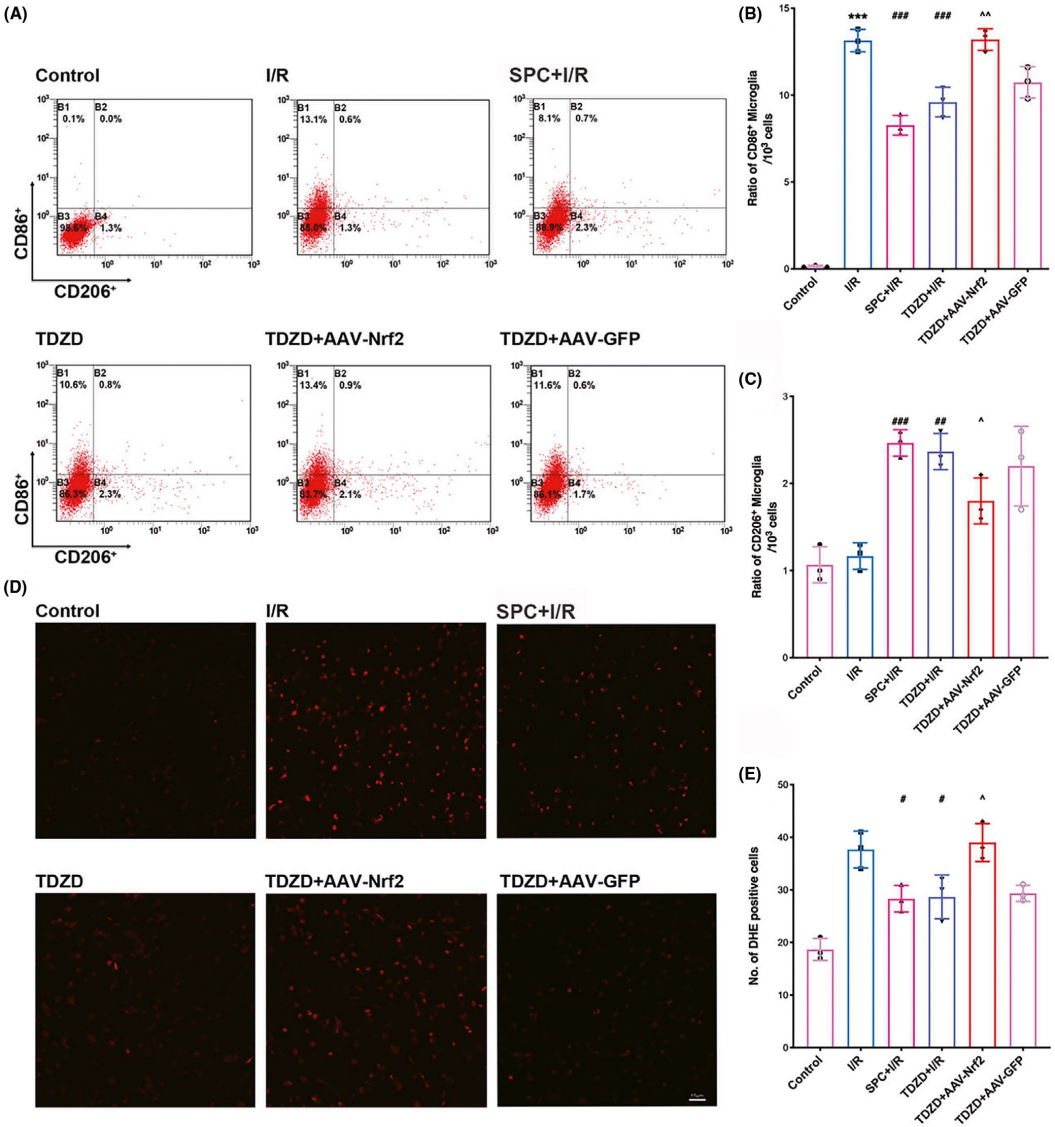
SPC and TDZD treatment alleviated pro‐inflammatory microglial but increased anti‐inflammatory microglial phenotype polarization and reduced the generation of ROS. These effects were reversed by the deficiency of Nrf2. (A) Flow cytometry analysis of CD86^+^ (pro‐inflammatory microglia) and CD206^+^ (anti‐inflammatory) cells in the ischemic penumbra 7 days after reperfusion. (B, C) The quantitative statistical results of CD206‐positive cells (B) and CD86‐ (C) in each group (*n* = 3). (D) Representative immunofluorescence micrographs showing DHE staining for ROS in each group. Scale bar = 15 μm. (E) The quantitative analysis of DHE‐positive cells. *p < 0.05, **p < 0.01, ***p < 0.001 versus the I/R group; #*p* < 0.05, ##*p* < 0.01, ###*p* < 0.001 versus the I/R group; ^*p* < 0.05, ^^*p* < 0.01, ^^^*p* < 0.001. One‐way ANOVA with Tukey's post hoc test was used for statistical analysis. AAV‐Nrf2, adeno‐associated virus induced Nrf2‐shRNA; DHE, dihydroethidium; I/R, ischemia/reperfusion; Sevo, sevoflurane preconditioning; TDZD, 4‐Benzyl‐2‐methyl‐1,2,4‐thiadiazolidine‐3,5‐dione

Representative images of flow cytometry used to examine the ratio of pro‐inflammatory/anti‐inflammatory positive microglia/macrophages are shown in Figure 8A. In Figure 8B, activated pro‐inflammatory microglia/macrophages (CD86^+^) intensely accumulated in the ischemic penumbra compared with the ipsilateral hemisphere of the control group (*p* < 0.001). Meanwhile, SPC and TDZD treatment significantly reduced this accumulation (*p* < 0.001 and *p* = 0.0003, respectively). However, this reduction was reversed by Nrf2 mutation (*p* = 0.003). In Figure 8C, the percentage of anti‐inflammatory ‐positive microglia/macrophages (CD206^+^) was not affected by I/R surgery, but both SPC and TDZD treatment increased this ratio compared with that of the I/R group (Sevo + I/R: *p* < 0.001, TDZD + I/R: *p* = 0.0013). As expected, supplementation with AAV‐Nrf2 reversed the accumulation of anti‐inflammatory positive microglia (*p* = 0.016). No significant difference was detected between the TDZD and TDZD + AAV‐GFP groups regarding the percentage of pro‐inflammatory and anti‐inflammatory positive microglia/macrophages.

As indicated in Figure 8D, the generation of reactive oxygen species (ROS) was measured by DHE staining. Increased levels of DHE‐positive cells were detected in the I/R group, while both SPC and TDZD treatment reduced this increase (Figure 6E, Sevo + I/R: *p* = 0.0257, TDZD + I/R: *p* = 0.0323). Moreover, Nrf2 knockdown reversed the reduction in DHE‐positive cells induced by TDZD (*p* = 0.0131).

## 4 DISCUSSION

Using an in vivo transient focal cerebral I/R model and in vitro OGD/LPS‐induced injury models, the current study demonstrated that GSK‐3β phosphorylation‐regulated Nrf2 activation was involved in the promoted anti‐inflammatory microglia/macrophages polarization produced by SPC after brain ischemia. SPC shifted microglia/macrophages polarization toward anti‐inflammatory phenotype, and increased the phosphorylation of GSK‐3β. Additionally, supplementation with the GSK‐3β inhibitor TDZD increased anti‐inflammatory microglia/macrophages shift and produced ischemic tolerance. Moreover, sevoflurane preconditioning or TDZD administration increased Nrf2 nuclear translocation, reduced the infarct volume, improved neurological function, attenuated cellular apoptosis, reduced the mRNA levels of pro‐inflammatory factors, decreased super‐oxidants generation, and promoted the mRNA expression of anti‐inflammatory factors after brain ischemia. However, these benefits induced by SPC or TDZD were reversed by the knockdown of Nrf2. Taken together, the results of this study identified a potential underlying mechanism of SPC‐induced neuroprotection against cerebral I/R injury.

Various preconditioning therapeutics, such as sevoflurane, elicit neuroprotective effects by attenuating inflammatory process‐related signals, including TNF‐α and nuclear factor κB.^31^, ^37^, ^38^, ^39^ Furthermore, a previous study demonstrated that hyperbaric oxygen preconditioning attenuated ICH‐induced brain damage by inhibiting pro‐inflammatory microglia phenotype polarization,^40^ indicating a potential association between microglia polarization and preconditioning stimuli. However, in the brain, the underlying mechanism of SPC's regulatory effect on the pro‐inflammatory/anti‐inflammatory microglia phenotype switching remains unclear. A considerable amount of evidence has shown that different microglia/macrophages phenotypes could be indicated by changes in the expression of some inflammatory cytokines. TNF‐α, IL‐1β, and iNOS are related to the pro‐inflammatory phenotype, while CD‐206, YM1/2, and arginase‐1 correlate with the anti‐inflammatory phenotype.^10^, ^41^, ^42^, ^43^, ^44^, ^45^ In the current study, we found that SPC induced a significant increase in CD‐206, YM1/2, and arginase‐1 but a noticeable reduction of TNF‐α, IL‐1β, and iNOS mRNA. Additionally, the current study also found that SPC increased Arg1‐positive microglia phenotype while reduced iNOS positive microglia/macrophages phenotype. These findings are consistent with previous studies identifying different roles of microglia phenotype in neurodegenerative diseases, including cerebral ischemic stroke.^46^ To elucidate the protective role of SPC against inflammation response‐related microglia apoptosis, we employed the OGD‐induced injury model and LPS‐stimulation model in primary cortical microglia culture. Results demonstrated that SPC also prevented pro‐inflammatory cytokines mRNA expression and enhanced the expression of anti‐inflammatory cytokines genes in these in vitro models, which was consistent with other studies using different protective stimuli.^12^, ^47^

Another major finding of this study is that SPC engages anti‐inflammatory microglia/macrophages phenotype polarization possibly in a GSK‐3β phosphorylation‐dependent manner. In addition to regulating physiological processes such as glucose metabolism, cellular development, and differentiation, the phosphorylation of GSK‐3β is also a target that prevents ischemic insult. In the CNS, GSK‐3β was expressed in both neurons and microglia, which affected the microglial/macrophages activation by modulating a cascade of signals.^48^, ^49^, ^50^, ^51^ SPC increased the phosphorylation of GSK‐3β in ischemic penumbra and also in primary microglia culture after OGD. We also employed TDZD, a GSK‐3β inhibitor at Ser9 that previously used by other groups, and found that both SPC and TDZD elicited a neuroprotective effect by improving anti‐inflammatory marker gene expression and reducing pro‐inflammatory marker mRNA expression.^52^, ^53^ These results indicated that SPC‐mediated improvement of anti‐inflammatory microglia/macrophages phenotype polarization was GSK‐3β inactivation dependent.

SPC protects the brain by GSK‐3β inhibitor‐enhanced anti‐inflammatory microglial polarization, but how GSK‐3β regulates this phenotype shift is still being debated. Previous studies by our group and others found that the Nrf2‐regulated antioxidant response element (ARE) participated in the neuroprotective and antioxidant effects of SPC. This study also revealed that SPC‐induced Nrf2 activation might be partly mediated by other Keap1‐independent mechanisms.^3^, ^54^ GSK‐3β has been reported to function as a supplement to the Keap1‐Nrf2 degradation mechanism and influence the nuclear exclusion and inactivation of Nrf2.^17^, ^23^ GSK‐3β has also been demonstrated to downregulate the Nrf2/ARE pathway and may represent a potential treatment target against cerebral I/R injury.^15^, ^17^, ^26^ In this study, we found that GSK‐3β inhibition, which was induced by TDZD or SPC, increased Nrf2 translocation, while the knockdown of Nrf2 by administrating AAV‐shRNA reversed the enhanced anti‐inflammatory microglia/macrophages phenotype shift and neuroprotective effect. This finding provides a plausible explanation for how GSK‐3β could regulate anti‐inflammatory microglia/macrophages activation after SPC. Additionally, considering the relationship between oxidative stress and inflammatory pathways and Nrf2's ability to regulate these signaling cascades, we found that the knockdown of Nrf2 reversed the anti‐inflammatory microglia/macrophages shift induced by SPC or TDZD, which is in consistent with the findings on super‐oxidants staining.

### 4.1 Limitations

Some limitations of the present study should be noted. First, we did not clarify the role of cell‐specific expression of Nrf2, especially in microglia, in the regulation of inflammasome or other inflammatory response produced by SPC.^55^ Moreover, microglia/macrophages appear to be heterogeneous with diverse functional phenotypes that range from immuno‐enhanced phenotypes to anti‐inflammatory phenotypes.^56^

Considering the unique roles of microglia/macrophages, the promotion of neurogenesis and angiogenesis produced by the activation of the immune‐suppressive microglia phenotype may play a vital part in stimulating neuroprotective mechanisms to protect neurons from injury.^12^, ^16^, ^47^, ^57^ However, whether these complex phenotypes of microglia/macrophages could be directly or indirectly affected by SPC, and whether this polarization of phenotypes was influenced by sex or sex related signaling, still needs to be studied in the future.^58^

## 5 CONCLUSIONS

In summary, this study demonstrated that GSK‐3β phosphorylation‐mediated Nrf2 activation is involved in SPC‐induced cerebral ischemic tolerance by shifting microglia/macrophages toward anti‐inflammatory phenotype after cerebral I/R. These investigations may reveal a potential mechanism of SPC‐induced neuroprotection.

## CONFLICT OF INTEREST

The authors declare that they have no competing interests.

## AUTHOR CONTRIBUTIONS

Min Cai, Shiquan Wang, and Wugang Hou designed and conducted the experiments, collected and analyzed the data, and prepared the paper. Sisi Sun, Jin Wang, Qianzi Yang, Li Tian, and Beibei Dong participated in the experiments confirmation, data collection, and analysis. Min Cai, Hailong Dong, and Wugang Hou supervised the study and helped with the study design and data analysis and completion of the manuscript. All authors approved the final manuscript.

## ETHICAL APPROVAL

All animal experimental procedures were approved by the Ethics Committee for Animal Experimentation of the Fourth Military Medical University and followed the National Institutes of Health Guide for the Care and Use of Laboratory Animals.

## Supporting information

Supplementary Material

## Data Availability

The data that support the findings of this study are available from the corresponding author upon reasonable requests.
