# Supplementary material for: Sevoflurane preconditioning protects experimental ischemic stroke by enhancing anti‐inflammatory microglia/macrophages phenotype polarization through GSK‐3β/Nrf2 pathway
Source: CNS Neurosci Ther. 2021 Aug 9;27(11):1348–65. doi: 10.1111/cns.13715 (PMC8504524; doi:10.1111/cns.13715)
Supplement: Supplementary file 1 — Supplementary Material [file CNS-27-1348-s001.docx]

**Supplementary Table 1. Data of arterial gas analysis in oxygen control group or sevoflurane preconditioning group.**

| Group | MAP | T(°C) | PH | PaO_2_(mmHg) | PaCO_2_(mmHg) |
| --- | --- | --- | --- | --- | --- |
| 5 min before MCAO |  |  |  |  |  |
| Control | 99.8.1±2.3 | 37.5±0.1 | 7.42±0.05 | 108.0±2.1 | 39.1±2.3 |
| I/R | 103.2±2.1 | 37.3±0.1 | 7.42±0.02 | 102.3±1.0 | 39.0±1.9 |
| SPC | 102.0±1.2 | 37.4±0.1 | 7.41±0.05 | 104.3±0.9 | 38.2±2.2 |
| 30 min after MCAO |  |  |  |  |  |
| Control | 101.2±2.1 | 37.3±0.3 | 7.39±0.06 | 104.3±0.9 | 39.5±1.8 |
| I/R | 102.0±1.9 | 37.0±0.2 | 7.39±0.06 | 109.1±0.8 | 39.6±2.2 |
| SPC | 102.8±1.8 | 37.2±0.2 | 7.41±0.05 | 106.5±1.3 | 38.7±2.4 |

Using separate mice, data for arterial gas were obtained before, during, and after MCAO operation in sevoflurane preconditioning and oxygen-inhaled groups. There were no significant differences between the two groups at the corresponding time points. All values are expressed as manorial. (n = 5 per group). All values are expressed as Mean ± SD.

SPC = sevoflurane preconditioning; MCAO = middle cerebral artery occlusion; I/R = ischaemia/reperfusion.

**Supplementary Figure 1. SPC didn’t alter the change of regional cerebral blood flow of ischemic hemisphere durin g MCAO surgery.**


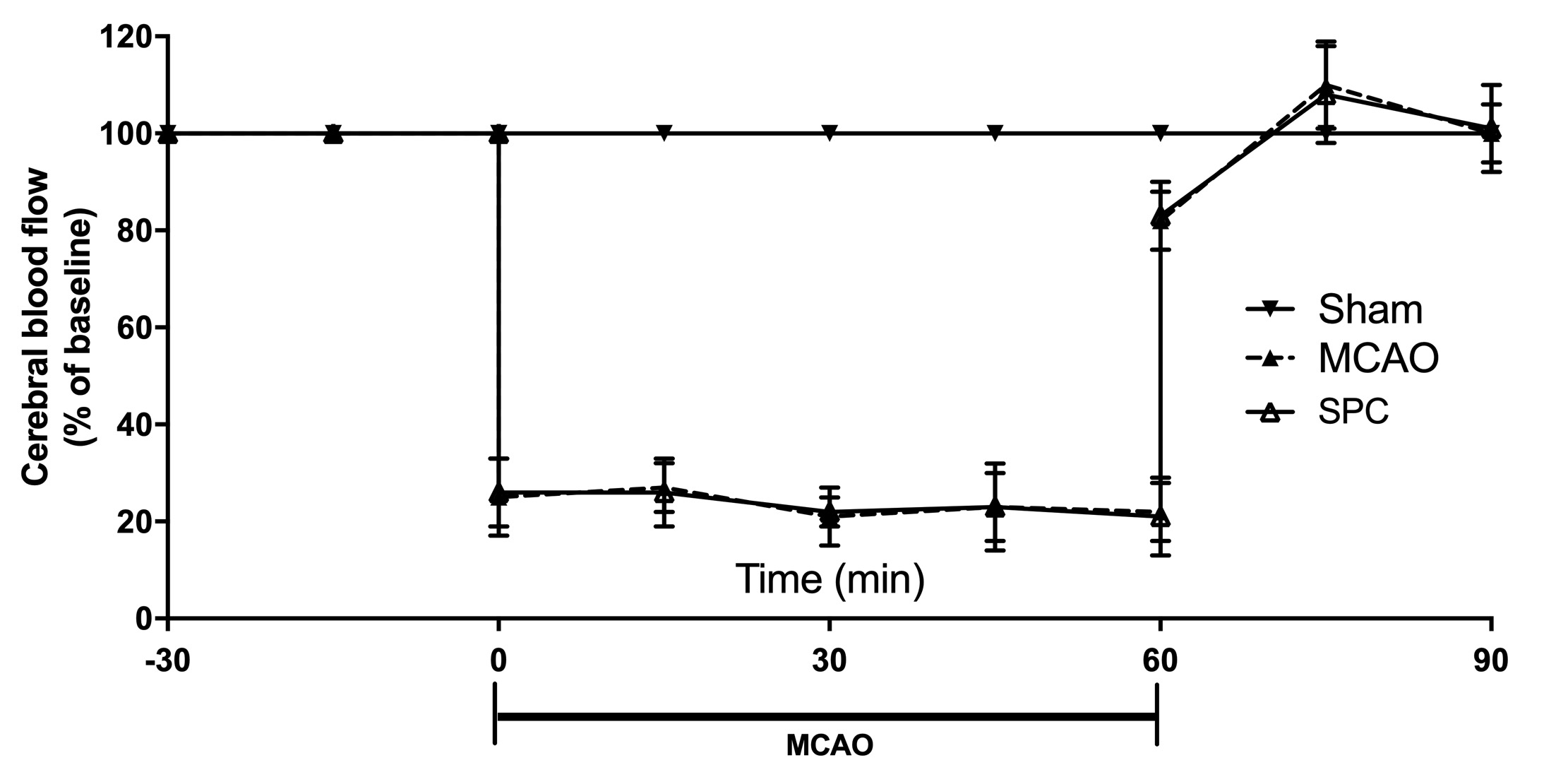


The sevoflurane preconditioning didn’t alter the changes of regional cerebral blood flow induced by MCAO surgery. n = 5 per group. SPC = sevoflurane preconditioning; MCAO = middle cerebral artery occlusion. One-way ANOVA with Tukey’s post hoc test was used for statistic analysis.

**Supplementary Figure 2. The expression of GSK-3β was measured by western blot.**





The phosphorylation of GSK-3β in nuclear was examined by western-blot. No significant difference was detected among I/ + SPC, I/R + TDZD and I/R + SPC + TDZD group. One-way ANOVA with Tukey’s post hoc test was used for statistic analysis.

**Supplementary Figure 3. The expression changes of Nrf2 after AAV-mediated siRNA supplementation.**





Nrf2 protein content was decreased about 45% in the penumbra-like area after AAV-Nrf2 microinjection, as compared to the control group (*P* = 0.016). The control AAV-GFP didn’t alter the expression of Nrf2.
